# The Crystal Structure of Peroxiredoxin Asp f3 Provides Mechanistic Insight into Oxidative Stress Resistance and Virulence of *Aspergillus fumigatus*

**DOI:** 10.1038/srep33396

**Published:** 2016-09-14

**Authors:** Falk Hillmann, Karine Bagramyan, Maria Straßburger, Thorsten Heinekamp, Teresa B. Hong, Krzysztof P. Bzymek, John C. Williams, Axel A. Brakhage, Markus Kalkum

**Affiliations:** 1 Junior Research Group Evolution of Microbial Interactions, Leibniz Institute for Natural Product Research and Infection Biology - Hans-Knöll-Institut, Beutenbergstrasse 11a, D-07745 Jena, Germany; 2 Department of Molecular Immunology, The Beckman Research Institute of City of Hope, 1500 East Duarte Road, Duarte, CA 91010, USA; 3 Transfer Group Anti-infectives, Leibniz Institute for Natural Product Research and Infection Biology - Hans-Knöll-Institut, Beutenbergstrasse 11a, D-07745 Jena, Germany; 4 Department of Molecular and Applied Microbiology, Leibniz Institute for Natural Product Research and Infection Biology - Hans-Knöll-Institut, Beutenbergstrasse 11a, D-07745 Jena, Germany; 5 Department of Molecular Medicine, The Beckman Research Institute of City of Hope, 1500 East Duarte Road, Duarte, CA 91010, USA; 6 Institute of Microbiology, Friedrich Schiller University Jena, D-07743 Jena, Germany

## Abstract

Invasive aspergillosis and other fungal infections occur in immunocompromised individuals, including patients who received blood-building stem cell transplants, patients with chronic granulomatous disease (CGD), and others. Production of reactive oxygen species (ROS) by immune cells, which incidentally is defective in CGD patients, is considered to be a fundamental process in inflammation and antifungal immune response. Here we show that the peroxiredoxin Asp f3 of *Aspergillus fumigatus* inactivates ROS. We report the crystal structure and the catalytic mechanism of Asp f3, a two-cysteine type peroxiredoxin. The latter exhibits a thioredoxin fold and a homodimeric structure with two intermolecular disulfide bonds in its oxidized state. Replacement of the Asp f3 cysteines with serine residues retained its dimeric structure, but diminished Asp f3’s peroxidase activity, and extended the alpha-helix with the former peroxidatic cysteine residue C61 by six residues. The *asp f3* deletion mutant was sensitive to ROS, and this phenotype was rescued by ectopic expression of Asp f3. Furthermore, we showed that deletion of *asp f3* rendered *A. fumigatus* avirulent in a mouse model of pulmonary aspergillosis. The conserved expression of Asp f3 homologs in medically relevant molds and yeasts prompts future evaluation of Asp f3 as a potential therapeutic target.

The opportunistic pathogen *Aspergillus fumigatus* is a filamentous fungus that is ubiquitously distributed throughout the environment. As a saprophyte it contributes to biomass recycling by decomposing decaying plant materials^1^,^2^. In hypersensitive individuals inhaled *A. fumigatus* spores (conidia) are responsible for allergic bronchopulmonary aspergillosis (ABPA), also known as farmer’s lungs. More importantly, *A. fumigatus* can cause severe debilitating and oftentimes lethal infections in immunocompromized patients. Invasive fungal infections such as invasive aspergillosis are predominantly caused by *A. fumigatus* and represent the prime obstacle to the success of hematopoietic stem cell transplantation in the cancer clinic^1^,^3^,^4^.

Reduced production of host derived reactive oxygen species (ROS), as in patients with chronic granulomatous disease (CGD), is regarded as one of the highest risk factors^5^, although the defensive role of ROS in fungal immunity is not fully understood. Reactive oxygen species generated by innate immune cells are components of the fundamental defense mechanism against infection with *A. fumigatus*, but whether these toxic molecules target the fungus directly is not known. Here, we have studied the *A. fumigatus* protein Asp f3 and its role for the protection of the fungus against ROS.

The intracellular Asp f3 is an abundant protein in *A. fumigatus.* Asp f3 was originally identified as a major fungal allergen with affinity to serum immunoglobulin (Ig)E from patients with ABPA^6^. However, it was also shown that Asp f3 can serve as a promising vaccine candidate^7^,^8^. Asp f3-immunized mice were protected from experimentally induced invasive pulmonary aspergillosis in both corticosteroid immunosuppressed and in neutrophil depleted mice^9^. In the fungus, Asp f3 co-localizes with peroxisomes and shows peroxide dependent up-regulation at the transcriptional and translational level^8^,^10^. It contains 168 amino acid residues and BLAST searches reveal considerable sequence homology to the thioredoxin superfamily, the peroxiredoxin (Prx) family, and the PRX5-like subfamily.

Here, we report the crystal structure of Asp f3 as a dimeric molecule with two interchain disulfide bonds. We experimentally confirm the predicted peroxiredoxin activity of Asp f3 and demonstrate the functional contribution of its cysteine residues for catalytic activity. We show that Asp f3 is indispensible for the protection of *A. fumigatus* against oxidative stressors. Finally, we provide evidence for the critical role of Asp f3 in fungal virulence, wherein it protects *A. fumigatus* against oxidative stress *in vitro*, and *in vivo*, in a murine model of invasive pulmonary aspergillosis.

## Results

### Asp f3 Structure Determination

**Figure 1:**
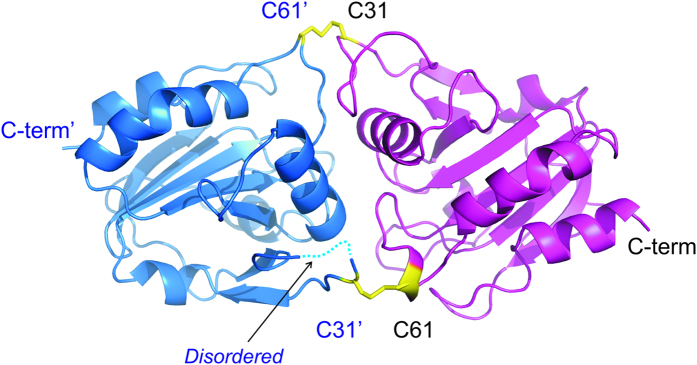
Ribbon cartoon of Asp f3 structure at 2.1 Å resolution. Asp f3 forms a homodimer, juxtaposing C31 in one protomer (magenta) and C61 in the other protomer (blue). Shown here is the oxidized form of Asp f3, generating two disulfide bonds across the dimer interface (yellow sticks).

**Figure 2:**
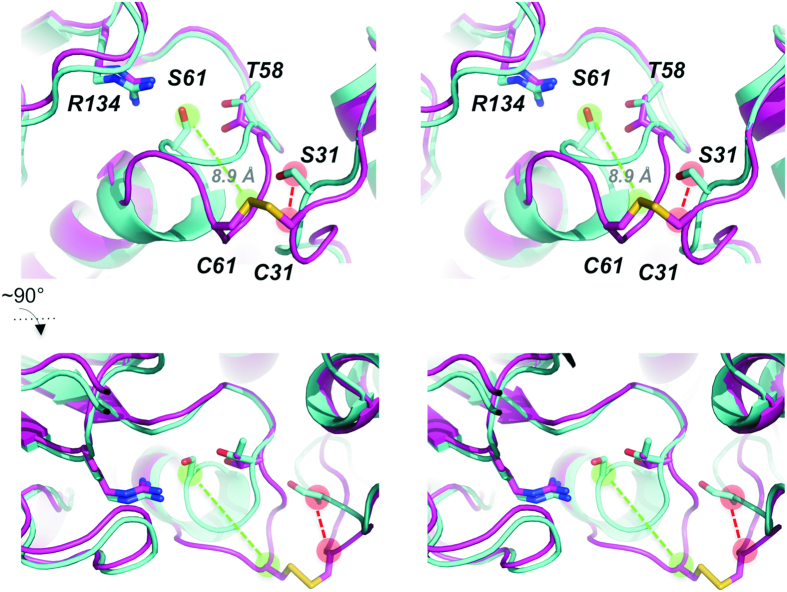
Superposition of WT Asp f3 dimer and C31S/C61S indicates a major conformational change upon oxidation. The structure of the C31S/C61S mutant (cyan), a mimic of the reduced Asp f3, was superimposed on WT Asp f3 (magenta). The backbone of each are represented as cartoons and the side chains of the conserved residues, C31, C61, T58 and R134, are represented as sticks. Shown in stereo, the top view (**a**) and the bottom view (**b**) differ by a 90° rotation to highlight the large conformational change between the reduced-state mimic and the oxidized state. The partial unwinding of the α-helix in the reduced state to the oxidized state produces a 8.9 Å shift of the C61 sulfhydryl group (light green circles). Concomitant with the repositioning of C61 is a small shift in C31 (light red circles).

Aspf3 wild type (WT) and its C31S/C61S variant crystallized as a homodimer and showed a typical thioredoxin-like fold^11^,^12^,^13^. The WT protein was captured in its oxidized state, with two intermolecular disulfide bonds across the dimerization interface between C31 and C61 of the individual protomers (Fig. 1, and Supplementary Fig. S1). Searches using DALI^14^ indicate that the structure is very similar to recently published structure of yeast peroxiredoxin Ahp1, PDB ID 4DSQ (RMSD of 1.4 Å)^15^. In the WT structure, both C31 and C61 reside in flexible loop regions (large B-factors and lack of well-defined electron density for one of the chains). The serine variant showed little overall structural change (RMSD 1.7 Å), with large deviation of the aforementioned loops (residues 24–35 and 58–64 RMSD > 4.0 Å). Disruption of the intermolecular disulfide bond in C31S/C61S variant extends the α-helix by 6 residues resulting in a significant movement of S61 by ~7–9 Å from the position occupied by C61 in the WT structure (Fig. 2, Table 1), analogous to the movement of C62 in the reduced form of Ahp1^15^. A corresponding movement of S31 by 4–5 Å is likely driven by formation of a hydrogen bond to backbone oxygen of F57. Further details regarding the inter-chain interactions of both structures are given in Supplementary Table S1.

Sedimentation equilibrium studies by analytical ultracentrifugation of WT Aspf3 and C31S/C61S proteins indicate that the dimerization is independent of the Cys oxidation state (Supplementary Fig. S2). Global fit analysis of sedimentation equilibrium experiments of WT Aspf3 and C31S/C61S revealed the presence of a dimer (SEDPHAT calculated molecular weight – 36 kDa and 37.3 kDa for WT and C31S/C61S, respectively; theoretical molecular weight – 38.6 kDa). Sedimentation equilibrium experiment performed on WT protein in the presence of excess TCEP revealed the presence of the dimer (calculated MW of 35.5 kDa). The Asp f3 C31S/C61S dimer, as well as the reduced WT Asp f3 separate primarily as monomers under the chaotropic conditions of SDS gel electrophoresis (Supplementary Fig. S3E).

### Biochemical evaluation of the Asp f3 peroxidase (Prx) activity

**Figure 3:**
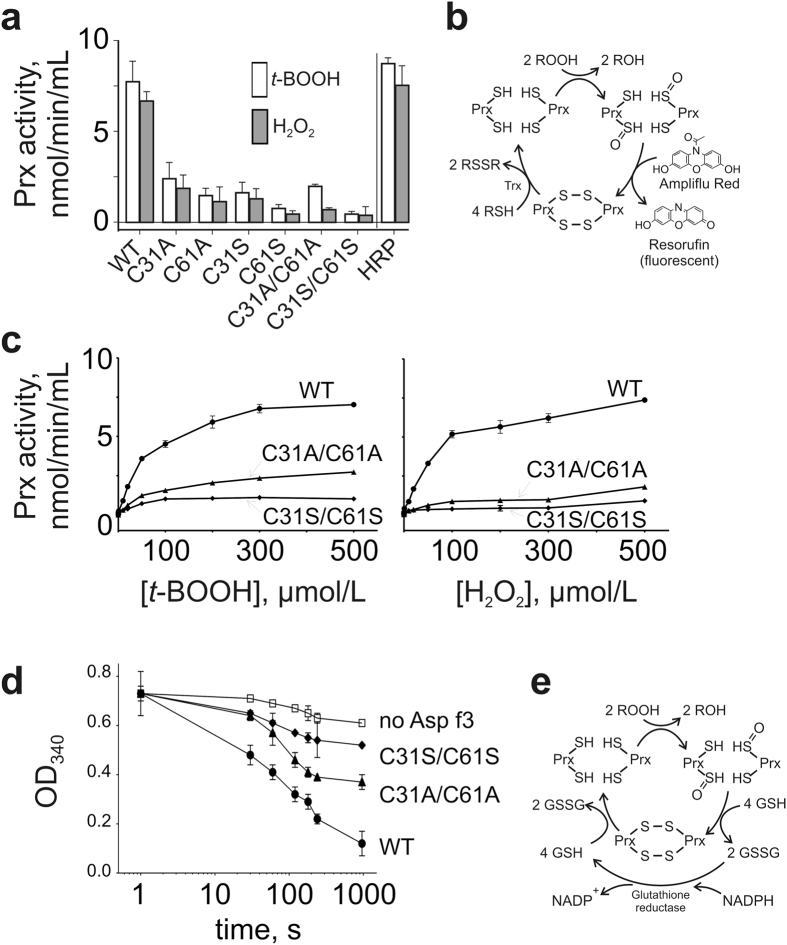
Prx activity of Asp f3 and Cys mutants. (**a**) Effect of cysteine replacements on Prx activity of Asp f3 measured in the presence of H_2_O_2_ or *t-*bOOH with Ampliflu Red. (**b**) Reaction schematics of the Ampliflu Red assay for Prx activity. (**c**) Reaction kinetics of Asp f3 and mutants measured in the presence of increasing concentrations of H_2_O_2_ or *t-*bOOH with Ampliflu Red. (**d**) NADPH-dependent Prx activity of Asp f3 and Cys replacement mutants determined in the presence *t-*bOOH, GSH, GSHR, monitored as a decline in NADPH light absorbance at 340 nm. (**e**) Reaction scheme of the NADPH-based Prx activity assay.

To confirm that Asp f3 indeed exhibits the predicted Prx activity, we conducted two independent biochemical assays on WT Asp f3, its cysteine/serine (Cys/Ser) mutants, and the corresponding cysteine/alanine (Cys/Ala) mutants. Direct assessment of Prx was conducted by measuring Prx-dependent conversion of 10-acetyl-3,7-dihydroxyphenoxazine (Ampliflu Red) to fluorescent 7-hydroxyphenoxazin-3-one (Resorufin) in presence of thioredoxin, hydrogen peroxide or *tert*-butyl hydroperoxide (*t-*bOOH) (Fig. 3a–c); Secondly, Prx activity was determined indirectly, by colorimetric evaluation of the NAPDH oxidation as a consequence of glutathione reductase activity which recycles Prx activity in presence of *t-*bOOH (Fig. 3d,e). Both Cys/Ser as well as Cys/Ala replacements in Asp f3 dramatically lowered its peroxiredoxin activity with *t*-bOOH and H_2_O_2_ (Fig. 3a,c). These observations indicated that C31 and C61 are involved in the catalytic mechanism of Asp f3.

Kinetic constants were determined for the WT and its cysteine double mutants (Table 2, Fig. 3c). Cysteine replacement decreased the enzymatic turnover rate (*k*_*cat*_) of the WT by 2.6 to 6.8-fold with *t*-bOOH, and 4 to 12-fold with H_2_O_2_ for C31A/C61A and C31S/C61S mutants, respectively (Fluorometric Assay, Table 2 and Fig. 3c). A three-fold reduction of the WT catalytic efficiency rate (*k*_*cat*_*/K*_*m*_) was determined for C31A/C61A, and a ten-fold reduction for C31S/C61S with *t*-bOOH or H_2_O_2_, respectively (Table 2). Asp f3-dependent oxidation of NADPH in presence of glutathione reductase (Fig. 3d,e) confirmed the aforementioned results obtained with Ampliflu Red, and the kinetic data were comparable (Table 2). In comparison to the WT, a significantly lower time-dependent decrease in absorbance at 340 nm due to the oxidation of NADPH was monitored with C31A/C61A at 49% and C31S/C61S at 19% in presence of *t*-bOOH (Fig. 3d).

### Asp f3 protects from oxidative stress

**Figure 4:**
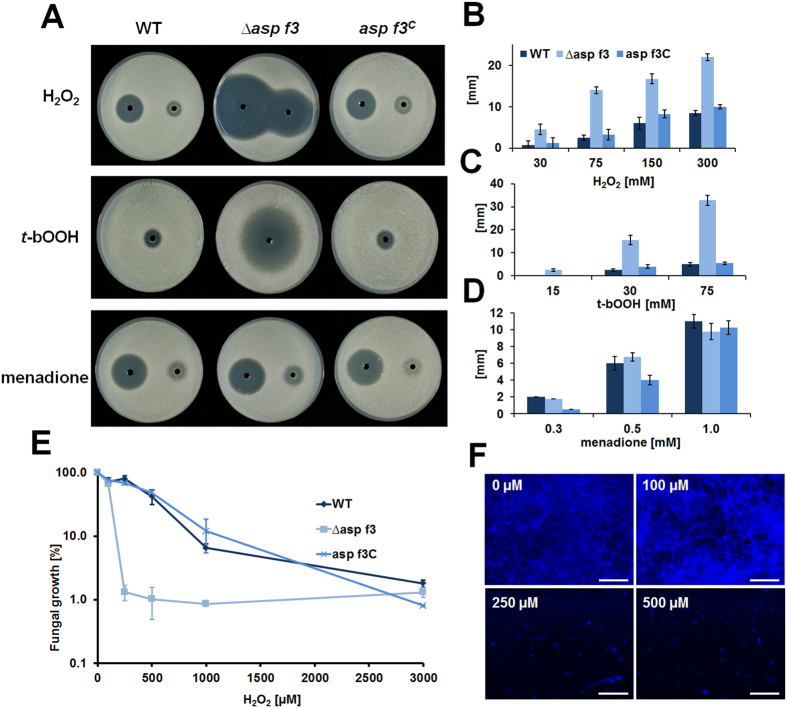
Asp f3 protects from peroxide stress. (**A**) Preinoculated conidia of *A. fumigatus* D141 (WT), the *asp f3* deletion mutant (∆*asp f3*) and the complemented strain *(asp f3*^*C*^) were grown for 18 h at 37 °C during agar diffusion of 50 μl of 300 mM (left) or 75 mM (right) H_2_O_2_, of 30 mM *t-*bOOH, and 1 mM (left) or 0.25 mM (right) of menadione. (**B–D**) Columns give the average size of inhibition zones (mm) with distinct concentrations of each stress factor determined for all strains in four biological replicates; error bars show standard deviation. (**E**) Growth of fungal strains as in (**A–D**) in the presence of different concentrations of H_2_O_2_. Values are expressed as average percentages of calcofluor white fluorescence relative to untreated controls in three biological replicates. (**F**) Fluorescence micrographs of ∆*asp f3* after 24 h of growth in the absence (0 μM) or presence of 100, 250, and 500 μM of H_2_O_2_ following chitin specific staining with calcofluor white. Bars are 100 μm.

Structural and functional evidence of Asp f3 as a Prx strongly suggested that Asp f3 could provide protection against oxidative stress *in vivo.* Southern and Western blot analysis confirmed the successful deletion of the *asp f3* gene and knock out (KO) of Asp f3 expression in *A. fumigatus* D141, as well as subsequent complementation of the mutant (*asp f3*^*C*^; Supplementary Fig. S3). The mutant was viable and growth was indistinguishable from the WT under standard growth conditions in *Aspergillus* Minimal Medium (AMM) at 37 °C. However, deletion of *asp f3* rendered *A. fumigatus* sensitive to H_2_O_2_ compared WT, revealed by agar diffusion assays revealed at concentrations exceeding 30 mM H_2_O_2_ (Fig. 4A,B). Expression of the *asp f3* genetic locus from an ectopic site of the genome (*asp f3*^*C*^) reverted peroxide sensitivities almost to WT levels. Peroxide sensitivity of the mutant was even more pronounced when using the organic hydroperoxide *t-*bOOH with a detectable inhibition zones at concentrations as low as 15 mM (Fig. 4C). In contrast, menadione which generates ROS through intracellular redox cycling^16^, was effective against *A. fumigatus* at less than 0.3 mM, but independent of the presence or absence of Asp f3 (Fig. 4D). To further define the *in vivo*-impact of the Asp f3 protein, we determined the minimal inhibitory concentrations for H_2_O_2_. During direct exposure in liquid cultures, *A. fumigatus* WT cells maintained growth at H_2_O_2_ levels of 1000 μM, while growth of ∆*asp f3* was fully inhibited at 250 μM (Fig. 4E,F).

### The asp f3 deletion mutant is hypersensitive to exposure to ·O_2_^*−*^

**Figure 5:**
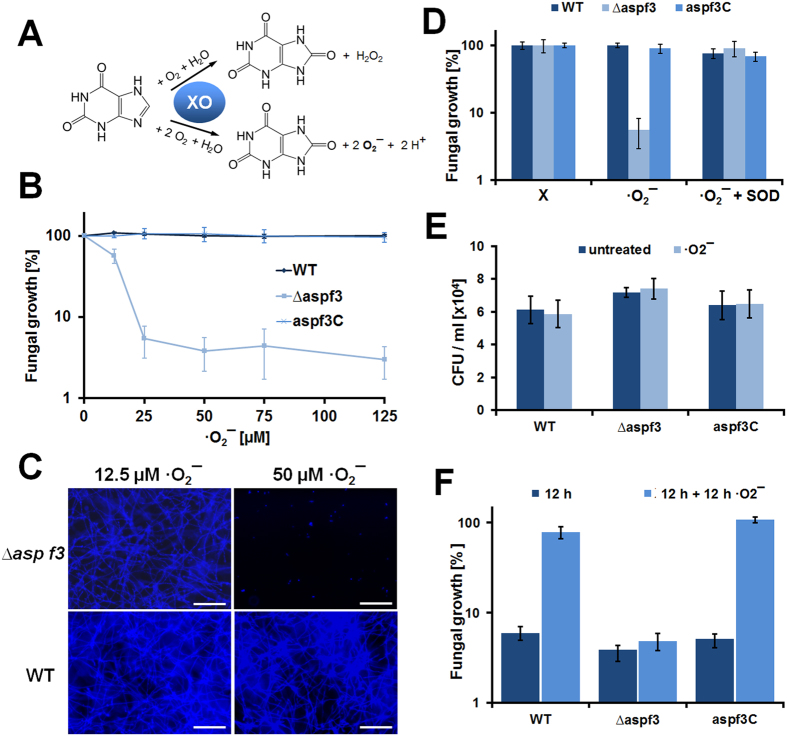
The *∆asp f3* mutant is hypersensitive to external superoxide. **(A**) Xanthine oxidase (XO) catalyzes the O_2_ dependent conversion of xanthine (X) to uric acid and generates a mixture of H_2_O_2_ and superoxide radicals (·O_2_^−^). (**B**) Swollen conidia of *A. fumigatus* D141 (WT), the *asp f3* deletion mutant (*∆asp f3*) and the complemented strain (*asp f3*^*C*^) were exposed to increasing concentrations of ·O_2_^−^. Fungal growth was monitored as total chitin formation as quantified by calcofluor white fluorescence. Values are expressed as average percentages of three biological replicates relative to the untreated controls (0 μM). **(C**) Fluorescence micrographs illustrating that fluorescence values were proportional to total mycelium formation and confirmed the different sensitivities of the *∆asp f3* and WT strain. White bars are 100 μm. (**D**) Growth inhibition of swollen conidia of *∆asp f3* was strictly dependent on the dual presence of X and XO and was abolished by addition of superoxide dismutase (SOD). (**E**) Colony forming units after a 2 h exposure of swollen conidia to X and XO. (**F**) ·O_2_^−^ dependent growth inhibition of hyphae: Conidia were grown into hyphae for 12 h (dark blue bars), hyphal growth monitored after an additional 12 h of cultivation in the presence of X and XO (bright blue bars).

Although *A. fumigatus* is likely to encounter the more stable H_2_O_2_ *in vivo*, ·O_2_^−^ represents the direct reaction product of NADPH oxidase complex (NOX) during interactions with host cells^17^. Hence, we tested whether extracellular ·O_2_^−^ would affect fungal growth and viability. The xanthin-oxidase (XO) reaction with xanthine (X) has previously been used as an alternative enzymatic system to generate this short-lived ·O_2_^−^ radical *in vitro* to assay bacteria or enzymes for their capability to scavenge and inactivate ·O_2_^− ^18,19,20. XO oxidizes X to uric acid with a concomitant release of ·O_2_^−^ and H_2_O_2_ (Fig. 5A). Under the reaction conditions used, xanthin oxidation released ·O_2_^−^ at 2:1 molar ratio based on the reduction of ferricytochrome *c* which was fully inhibited in the presence of an externally added superoxide dismutase^20^ (Supplementary Fig. S4). Conidia of the WT and the *asp f3*^*C*^ strain grew normally over the whole concentration range of added X, from 25 to 250 μM, corresponding to ·O_2_^−^ levels of 12.5 to 125 μM, respectively. In contrast thereto, the growth of the *∆asp f3* strain was drastically diminished by the X + XO treatment (Fig. 5B,C). This growth defect of the mutant was fully reverted either by the reintroduction of a functional copy of the *asp f3* gene (*asp f3*^*C*^) or the addition of external superoxide dismutase (SOD, Fig. 5D). The toxic effect of external ·O_2_^−^ was fungistatic rather than fungicidal as swollen conidia remained viable even after an exposure of 2 h, as revealed by CFU counts (Fig. 5E). ·O_2_^−^ levels as low as 12.5 μM were sufficient to reduce hyphae formation below 60% of that of the untreated controls. ·O_2_^−^ concentrations >25 μM were completely inhibitory for the growth of the *∆asp f3* strain, even when pre-grown to its hyphal form (Fig. 5F), giving direct evidence that the *in vivo* function of Asp f3 is essential to protect against low levels of extracellular ·O_2_^−^.

### Asp f3 is required for *A. fumigatus* virulence in experimental pulmonary aspergillosis

**Figure 6:**
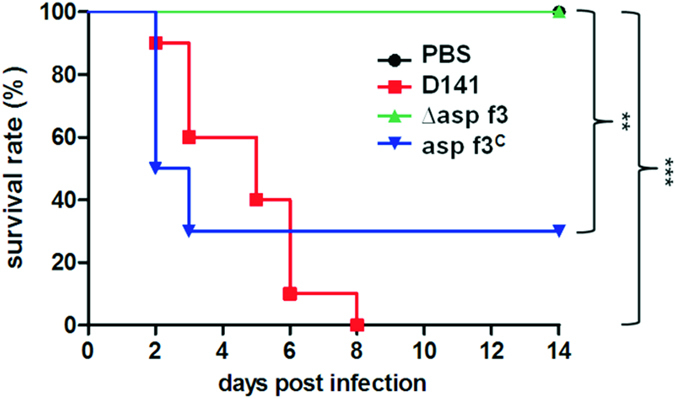
Asp f3 is required for *A. fumigatus* virulence in invasive aspergillosis. For each strain 10 mice were immunosuppressed with cortisone acetate and intranasally infected with conidia of the WT, the *asp f3* deletion strain (∆asp f3), and the complemented strain (asp f3^C^). A control group of mice was treated with PBS as a negative control. The graph shows the percentage of survivors at each day after infection. The number of asterisks indicate significances in log rank tests with p-values of p < 0.0012 (**) or p < 0.0001 (***).

The Prx activity of Asp f3 detected in our *in vitro* studies as well as its essential role in oxidative stress resistance *in vivo*, prompted us to test if Asp f3 could play a role during pulmonary invasive aspergillosis. Therefore, four groups of mice were immunosuppressed with cortisone acetate and animals remained uninfected (PBS-control) or were either infected with conidia of the WT, *∆asp f3*, or *asp f3*^*C*^ strain of *A. fumigatus* (Fig. 6). Animals infected with WT conidia died over a course of 8 days and histological analysis revealed severe signs of pulmonary aspergillosis (IA), including tissue invasion of fungal hyphae (Supplementary Fig. S5) and massive infiltration of inflammatory cells, including polymorphonuclear leukocytes (Supplementary Fig. S5). In contrast, all animals infected with conidia of the *∆asp f3* strain survived for the entire 14 day observation period without showing any clinical signs of the disease. When infected with conidia from the complemented strain *asp f3*^*C*^, IA developed at frequencies comparable to that of the WT and was lethal for 70% of the animals. Histological examinations of the mice surviving infection with the *∆asp f3* strain revealed only residual fungal growth without invasion of the peribronchial tissue and only a small number of inflammatory cells (Supplementary Fig. S5). The same mice showed occasional signs of goblet cell hyperplasia (Supplementary Fig. S5). In summary, infections with the *asp f3* deletion strain did not lead to IA, indicating the requirement of the Asp f3 Prx for *A. fumigatus* virulence in the immunosuppressed animal model.

## Discussion

Based on sequence homology and structural information, Asp f3 was identified as a member of the 2-Cys Prx5 subfamily of the thiol-dependent peroxidase family of proteins that is similar to human Prx5 (PDB ID: 1HD2). The latter is a homodimeric Trx peroxidase that exclusively exists as an A-type dimer (in contrast to all other Prxs)^21^. Prx5 is widely expressed in tissues and found in mitochondria, peroxisomes and in the cytosol that are major sources of reactive oxygen species (ROS). This protein is able to reduce hydrogen peroxide and alkyl hydroperoxides with the use of reducing equivalents derived from thiol-containing donor molecules such as thioredoxin and glutathione, and known to be functionally implicated in antioxidant protective mechanisms^21^. This suggests that Asp f3 may have an important role in peroxide detoxification mechanism.

The crystal structure of Asp f3 revealed its homodimeric configuration that includes two intermolecular disulfide bonds (Fig. 1). The dimer interface comprises an area of ~710 Å^2^ and ~730 Å^2^ for WT and C31S/C61S variant, respectively. The slight increase in the overall dimer interface area for C31S/C61S (~730 Å^2^) is due to the fact that the loop connecting residues 24–31 is ordered in both protomers, whereas the backbone cannot be traced in one of the protomers in the WT structure. Salt bridges between R131 and D91 from the two protomers and additional hydrogen bonds between main chain atoms (T29-V60, A30-V60, A30-C61), as well as residues involved in hydrophobic interactions (I28, A30, F57, P59, V60, V94, Y93, A97) contribute to the extensive interface interactions. The WT, as well as C31S/C61S variant, exhibited close similarity to oxidized and reduced forms of Ahp1, respectively^15^. S61 in C31S/C61S of Asp f3 becomes more buried, much like C62 in the reduced form of Ahp1. Therefore, we expect that the structure of the C31S/C61S mutant to bear close resemblance with that of the reduced dimeric Asp f3 WT.

Each monomer contains the PGAFTPVC redox center motif that defines a catalytic triad of Pro, Thr, and Cys. The PxxxTxxC active site motif is highly conserved in Prx5 proteins^22^ (Supplementary Fig. S6), wherein the cysteine sulfur is coordinated by Thr (Ser) and Arg. The residue at the active site (C61) is the peroxidatic cysteine (C_P_) that initiates the peroxide reduction by nucleophilic attack, when present in its thiolate form. Deprotonation of the C_P_ is facilitated by H-bonding with the threonine hydroxyl group and the positive charge of the arginine guanidinium group (Fig. 2). This is followed by generation of the sulfenic acid form of C_P_ and the alcohol product (ROH) of the reduced peroxide substrate (Fig. 3b). Indeed, our C61A or C61S mutations drastically reduce the Asp f3 Prx activity in presence of *t*-bOOH or H_2_O_2_ (Fig. 3a). The second Cys residue (C31) is therefore designated as the resolving cysteine (C_R_) that participates in catalysis in later reaction step. Disulfide reductases such as a thioredoxin or glutaredoxin return oxidized Asp f3 to its activated, reduced state.

Our data are consistent with a previous report on an Asp f3 homologue, the 2-Cys yeast peroxiredoxin Ahp1^15^. Although Ahp1 and Asp f3 share only about 37% sequence identity, the positions of C_P_ and C_R_ are conserved and both located in the same sequence orientation with C_R_ closest to the N-terminus (Fig. S5). The dimeric interface of Asp f3 is similar to that of Ahp1, but it differs at several key positions. First, N90 and D91 in Asp f3 are reversed in Ahp1 (Supplementary Fig. S6). In Asp f3, the side chain of D91 caps α-helix 2 (Supplementary Fig. S7). To compensate for the charge, R131 from the other Asp f3 protomer makes a salt bridge to the side chain carboxylate (distance = 3.0 Å). The equivalent residue in Ahp1 is a tryptophan. In addition, Y89 in Asp f3 in the same protomer makes a hydrogen bond to R131 (3.3 Å, Supplementary Fig. S7). The equivalent residue in Ahp1 is a valine. Noteworthy, this part of the interface is not affected by the large conformational change that involves the redox active cysteines. Additional compensatory changes occur throughout the structure, preserving the fold and dimeric interface while reducing the overall sequence identity.

Asp f3’s enzymatic activity on peroxides and structural similarity to the previously characterized yeast-orthologue Ahp1, suggested that Asp f3 could counteract oxidative challenges in this fungal pathogen. Asp f3-like proteins are also highly conserved within the Aspergilli. For example Asp f3 from *A. fumigatus* shares 90% amino acid identity with its homologue in *A. nidulans*. The latter is a Prx5-like protein (Uniprot accession number Q5ASN8), which was identified as a major thioredoxin binding protein, and is under transcriptional control of the CCAAT binding complex that coordinates the oxidative stress response in eukaryotes^23^,^24^.

ROS production is a central element of the host immune response and a severe hereditary defect such as CGD represents a high-risk for IA with incidence rates ranging from 20–40%^5^. Patients suffering from CGD share mutations in components of the NADPH oxidase (NOX) complex which lead to a reduced production of superoxide radicals ·O_2_^−^ and hence, compromise the microbiocidal activity of phagocytes. Indeed we demonstrated *in vitro* that fungistasis of the *asp f3* mutant was observed at 25 μM of X corresponding approximately to 12.5 μM of ·O_2_^−^. Intriguingly, this is within the concentration range predicted for phagocytic cells^25^, indicating that NOX derived ·O_2_^−^ could directly affect the redox homeostasis of *A. fumigatus.* Such conclusion is in line with a previous study which reported that inhibition of the fungal thioredoxin-1 facilitates killing during interactions with neutrophil granulocytes^26^. Consequently, we expected that such ·O_2_^−^ dependent fungistasis should also reduce the virulence of *A. fumigatus* when facing a partially intact innate immune system. Corticosteroid-mediated immunosuppression of patients represents a high risk factor for fungal infections and is widely used in mouse models of IA. Upon infection with *A. fumigatus* corticosteroid-treated mice are characterized by high neutrophil counts and a massive increase in the release of myeloperoxidase^27^. Despite histological evidence of viable fungus in the murine airways, none of the mice infected with the *asp f3* deletion mutant died over the course of the experiment, indicating that the fungal ROS hypersensitivity contributed to reduced virulence. This conclusion is consistent with ROS mediated damage to be attributed to the killing capacity of innate immune cells^28^,^29^. Therefore, it will be interesting to further elucidate the role of Asp f3 in host pathogen interactions. Future research may consider targeting the Prx activity of Asp f3 for therapeutic purposes.

## Materials and Methods

### Materials

Chemicals and media were from Sigma, Saint Louis, MO, unless otherwise indicated.

#### Recombinant Asp f3, WT, mutants, expression and purification

Recombinant N-terminally His-tagged Asp f3 was produced earlier in our laboratory^7^. However, we were unable to crystallize it. Therefore, we generated another version of the protein, this time with the His tag at the C-terminus. In brief, we PCR amplified the Asp f3 insert from the original pMK2Aspf3 plasmid^7^ and cloned it into a pET28 vector (Novagen, EMD Biosciences, Madison, WI) using primers WTf and WTr (Supplementary Table S2) that encoded NcoI and NotI sites, respectively. Cysteine mutants were obtained with the QuikChange II XL Site-Directed Mutagenesis Kit (Agilent, Santa Clara, CA) using primers C31Af, C31Ar, C61Af, C61Ar, C31Sf, C31Sr, C61Af, and C61Ar (Supplementary Table S2). The resulting constructs were then transformed into the TOP10 competent cells (Thermo Fisher Scientific, Rockford, IL). Asp f3 was expressed in ClearColi BL21(DE3) *E. coli* cells (Lucigen, Middleton, WI) using 1 L terrific broth (BioPioneer, San Diego, CA) in presence of kanamycin (25 μg/mL), induced with isopropyl β-D-1-thiogalactopyranoside (IPTG) (0.1 mM), and cultured at 25 °C on an orbital shaker at 220 rpm for 16 hours. The bacteria were pelleted at 2000 × g and lysed with B-PER Complete Bacterial Protein Extraction Reagent (Thermo Fisher Scientific, Rockford, IL) containing 1× Halt Protease Inhibitor Cocktail (Thermo Fisher Scientific) and 10 units of Deoxyribonuclease I (Thermo Fisher Scientific). 10 mL lysate were passed through a 10-mL gravity flow column (Thermo) containing HisPur Ni-NTA resin (1 mL, Thermo), washed with 20 mL wash buffer (NaCl – 300 mM, Tris HCl – 20 mM, imidazole – 40 mM, at pH 7.4), and eluted with a wash buffer-based elution buffer (20 mL) in which the imidazole concentration was raised to 400 mM. The eluted protein was buffer exchanged by ultrafiltration on a YM-10 Centricon (Millipore) into FPLC buffer (NaCL–150 mM, Tris HCl – 20 mM, pH 8.0), and finally separated by size-exclusion chromatography on an ÄKTA purifier with a HiLoad Superdex 75 PG column (GE Healthcare, Pittsburgh, PA).

#### Crystallization, data collection, and structure determination

Initial crystallization conditions were identified using Microlytic’s MCSG-1 suite (Microlytic North America, Burlington MA) by vapor diffusion. Diffraction quality crystals of WT and cysteine variants of Aspf3 (both at 20 mg/ml) were obtained by vapor diffusion by mixing 1 μL of protein and 1 μL of the mother liquor (100 mM MES, pH 6.5, 25 mM NaCl and 27% [w/v] PEG3350, or 100 mM Tris, pH 7.2, 300 mM MgCl_2_ and 22% [w/v] PEG3350, for WT and C31S/C61S, respectively). The crystals were quickly wicked through the mother liquor containing 15–20% [w/v] meso-erythritol and frozen in the nitrogen stream of the diffractometer at 100 K. Diffraction data was collected at 1.5418 Å on the home source, a Rigaku MicroMax-007HF with R-Axis IV++detector. Diffraction data was processed and reduced using XDS, XSCALE and XDSCONV^30^. A Blast search against PDB database suggested that thioredoxin reductase from *B. cenocepacia* (PDB ID: 4F82) could serve as a potential model for molecular replacement (Blast E-value = 5 × 10^−29^). After failing to find a solution using the entire 4F82 model, flexible loops were removed and initial phases were obtained using Molrep^31^. Model rebuilding was done in Coot^32^. The refinement, and simulated annealing composite omit maps were performed in PHENIX^33^.

The crystallized recombinant Asp f3 contains an additional glycine residue in position 2 and a hexahistidine tag in position 170–175. Despite this and to simplify comparisons with native Asp f3, we are using the residue numbering of the published *A. fumigatus* Asp f3 sequences, NCBI reference accession numbers XP_747849, KEY83680, XP_747849, KMK55366, and EDP47753, in this publication. Both structures have been deposited at RSCB PDB (http://www.rcsb.org). PDB structure identifiers are 5J9B for WT Asp f3 and 5J9C for the C31S/C61S mutant.

#### Analytical ultracentrifugation

Protein samples (WT Aspf3 and C31S/C61S variant) were dialyzed overnight into phosphate-buffered saline (PBS), pH 7.4 (Slide-A-Lyzer MINI, 7000 MWCO, Thermo Fisher Scientific). Protein concentrations for the experiment were adjusted to optical density at A_280_ ~0.2, ~0.5 and ~0.8 (corresponding to ~5, ~15 and ~25 μM). To reduce disulfide bonds, a sample of WT protein in 1 mM TCEP in PBS was also analyzed. Sedimentation equilibrium experiments were performed using Beckman XL-I Proteomelab at 10000, 20000, and 30000 rpm, at 25 °C. Data was analyzed using Sedfit and Sedphat^34^.

#### *A. fumigatus* culture conditions

All fungal strains were stored in 20% (v/v) glycerol at −80 °C as conidia suspensions. These glycerol stocks were used to inoculate agar plates with AMM^35^. Conidia were harvested from surface culture grown for 5 d at 37 °C by rinsing with 0.1% [v/v] Tween-80. Fresh conidial suspensions were used to inoculate any liquid cultures at 10^3^–10^5^ conidia per mL and cultivated at 37 °C in either 24-well tissue culture plates (VWR International, Leuven, Belgium) or Erlenmeyer flasks on a rotary incubator at 200 rpm.

#### Asp f3 gene deletion and complementation

*A. fumigatus* D141 is a clinical isolate and was a gift from Utz Reichard, Universitätsmedizin Göttingen, Germany^36^. Deletion of the *asp f3* gene in *A. fumigatus* D141 was achieved by homologous recombination using the shuttle plasmid pAspf3-del. For construction of the plasmid, the two flanking chromosomal regions of the *asp f3* gene were amplified by nested PCR using primer AspF3-1 to AspF3-8 (Supplementary Table S2) and cloned into pBluescript II SK(+). A DNA cassette delivering hygromycin resistance was obtained from pME3002 and was inserted between both flanking regions using SfiI restriction sites^37^. For complementation the 2.7 kbp fragment covering the *asp f3* gene with 1600 bp and 250 bp of the upstream and downstream regions, respectively, was amplified and cloned into pSK275 (a kind gift from Sven Krappmann, Friedrich-Alexander-University of Erlangen-Nürnberg, Germany) using XmaI restriction sites and conferring resistance to the antimetabolite pyrithiamine. Plasmids were transformed into *A. fumigatus* using protoplast-transformation as described^35^. Transformants were either selected for resistance against hygromycin (250 μg/mL, Invivogen, Toulouse, France) or pyrithiamine (0.1 μg/mL, Sigma-Aldrich, Taufkirchen, Germany) and conidia underwent at least two successive rounds of selection to avoid heterokaryon contamination. Chromosomal integrations in the *asp f3* deleted strain (Δ*asp f3*) and the complemented strain (*asp f3*^*C*^) were verified by Southern hybridizations.

#### Analysis of Asp f3 protein expression

*A. fumigatus* D141, Δ*asp f3*, and *asp f3*^*C*^ strains were grown in PD medium at 37 °C for 24 h. The fungal material was separated from culture supernatant by centrifugation at 5,000 g. Proteins were extracted by sonication in presence of protease inhibitor cocktail, and then separated by SDS polyacrylamide electrophoresis (PAGE). 0.5 μg total protein (from ~10 mg dry hyphae) was loaded per lane and recombinant Asp f3 served as a positive control. Protein gels were stained with SimplyBlue SafeStain (Thermo Fisher Scientific) or used for Western blots developed with a previously described rabbit polyclonal anti-Asp f3 antibody and the IRDye 680RD donkey-anti-rabbit antibody (Li-Cor) as the secondary antibody^8^. The blots were scanned with a Li-Cor Odyssey infra-red Imaging System (Li-Cor Biotechnology, Lincoln, NE).

#### Isolation of chromosomal DNA from *A. fumigatus* and Southern Hybridization

DNA was isolated from overnight liquid cultures of *A. fumigatus*. The mycelium was separated from the medium by filtration, frozen in liquid N_2_ and ground to a fine powder in a mortar. Genomic DNA was extracted using the MasterPure yeast DNA purification kit (Epicenter Biotechnologies, Madison, WI, USA). For Southern hybridizations, restriction enzyme digested genomic DNA was separated on 1% (w/v) agarose gels and blotted onto Hybond-N+ membranes (GE Healthcare Europe, Freiburg, Germany). Blotting and hybridization procedures followed standard protocols^38^. Digoxigenin (DIG)-labeled probes were generated by adding DIG-11-dUTPs (Jena Bioscience GmbH, Jena, Germany) to the nucleotide pool of PCR reactions and were directed against a 430-bp fragment of the upstream region of the *asp f3* gene. Hybridization signals were detected using CDPstar^®^ as a highly sensitive chemiluminescent substrate of alkaline phosphatase coupled to an Anti-DIG-antibody (Roche Applied Science, Mannheim, Germany).

#### Effect of oxidative stress on *A. fumigatus’* viability

Fungal resistance to oxidative stress was assessed by exposure to various oxidative stressors. Resistance to high levels of oxidative stress was tested in agar diffusion assays by adding hydrogenperoxide (H_2_O_2_) *tert*-butyl hydroperoxide solution (*t-*bOOH) or menadione in varying concentrations (50 μL aliquots) to a central cavity of agar plates pre-inoculated with 10^5^ conidia of *A. fumigatus*. Diameters of inhibition zones were measured 18 h after incubation at 37 °C. The data displayed are mean values from four biological replicates and error bars indicate standard deviations. Lower concentrations of the oxidative stressors were required for the direct exposure assays to determine the minimal inhibitory concentration for H_2_O_2_, *t-*bOOH, and *in situ* generated superoxide (O_2_^−^). Conidia (10^5^ per mL) were transferred into liquid Czapek Dox medium (BD, Franklin Lakes, NJ) and distributed onto 24-well tissue culture plates (VWR International, Leuven, Belgium) in a final volume of 500 μl and cultured at 37 °C. After 6 h, H_2_O_2_ or *t-*bOOH were added to the cultures in varying concentrations. The xanthine-oxidase enzymatic system was used to generate O_2_^−^ *in vitro*^18^,^19^. Xanthine was added to each well up to final concentration of 250 μM, and the reaction was started by addition of 100 μg/mL (0.2 units/mL) xanthine oxidase (Sigma X4500). Where indicated, wells were also containing superoxide dismutase at 20 units/mL (Sigma S4636). After an additional 18 h, total cell wall chitin was determined as an indirect measure for fungal growth by staining with Calcufluor White (Fluorescence brightener 28, Sigma) at a final concentration of 0.1 mg/mL. After 5 min of incubation, plates were washed three times and total fluorescence was measured in an Infinite^®^ 200 PRO fluorometer (TECAN, Männedorf, Switzerland) at 360 nm (excitation) and 440 nm (emission). Data displayed are based on three biological replicates and with standard deviations given as error bars.

#### Peroxiredoxin (Prx) activity enzymatic assays

Prx activity was measured with a fluorometric assay that detected the reduction of H_2_O_2_ or *t-*bOOH in the presence of *E. coli* thioredoxin (Trx) and Ampliflu Red (10-acetyl-3,7-dihydroxyphenoxazine). Ampliflu Red stock solutions were prepared in DMSO at 2 mg/mL, aliquoted and stored at −20 °C. The reaction mix (100 μL) contained a series of dilutions of H_2_O_2_ or *t-*bOOH (1 nM to 500 μM) and Ampliflu Red (2 μg/mL) in 50 mM sodium phosphate (pH 7.4). WT Asp f3 or mutants were used at 5 μM final concentrations. Horse radish peroxidase (HRP), 10 pM, served as a positive control, and was also used to generate standard curves with H_2_O_2_ or *t-*bOOH substrates (Supplementary Fig. S9). The time-dependent fluorescence of the reaction product was measured using 535/585 nm excitation/emission wavelengths on a SpectraMax M2 fluorometer (Molecular Devices, Sunnyvale, CA) (Supplementary Fig. S8). Pre-reduction of peroxiredoxins or thioredoxin was performed with DTT (100 mM) for 1 hour at 22 °C as recommended in ref. 39. DTT was then removed from the sample using 3 kDa MWCO Vivaspin 500 centrifugal filter units (BioExpress, Kaysville, UT). Prx activity was calculated using the H_2_O_2_ or *t-*bOOH standard curve generated with HRP (Supplementary Fig. S9). The method was validated by an alternative photospectrometric assay that measures reduced nicotinamide adenine dinucleotide phosphate (NADPH)-dependent Prx activity in presence of reduced glutathione (GSH), glutathione reductase (GSHR), and *t-*bOOH^39^. Kinetic measurements were performed using 100 μL of reaction mix containing potassium phosphate (25 mM), pH 7.0, EDTA (1 mM), GSH (5 μM), GSHR (0.5 μM) and *t-*bOOH (100 μM). NADPH was added at the final concentration of 150 μM after which the absorbance at 340 nm was monitored for 20 min in presence or absence of Asp f3 protein and mutants. All assays were performed in triplicate, with error bars indicating standard deviation. Data analysis was performed with Prism 6.0 software (GraphPad software, La Jolla, CA).

#### Murine aspergillosis model

Pathogen-free female outbreed CD-1 mice (18–20 g, 6–8 weeks old, Charles River, Germany) were housed under standard conditions in individually ventilated cages, and fed with normal mouse chow and water *ad libitum*. Mice were immunosuppressed with two single doses of 25  mg cortisone acetate (Sigma Aldrich) suspended in PBS, injected intraperitoneally (i.p.) 3 days before and immediately prior to infection with conidia on day 0. To establish fungal infections, mice were anaesthetized by i.p. injection with midazolam (5 mg/kg), fentanyl (0.05 mg/kg), and medetomidine (0.5 mg/kg), and a 20 μL suspension of conidia (2 × 10^5^) in PBS were applied to the nares. Deep anesthesia ensured inhalation of the conidial inoculums. Anesthesia was terminated by subcutaneous injection of flumazenil, naloxon and atipamezol as described^40^. Mouse group sizes were n = 10 (infected strains) an n = 3 (PBS controls). Infected animals were monitored twice daily for weight loss, and moribund animals were sacrificed humanely. Fixation, Periodic acid-Schiff (PAS) and methenamine silver staining for histopathological analysis of the lungs of sacrificed mice was carried out as previously described^41^.

#### Ethics Statement

Mice were cared for in accordance with the principles outlined by the European Convention for the Protection of Vertebrate Animals used for Experimental and other Scientific Purposes. (http://www.coe.int/en/web/conventions/full-list/-/conventions/treaty/123). All experiments involving animals were in compliance with the German animal protection law and were approved by the responsible Federal State authority “Thüringer Landesamt für Verbraucherschutz (TLV)” and ethics committee “Beratende Komission nach § 15 Abs. 1 Tierschutzgesetz” with the permit Reg.-Nr. 03-001/12.

## Additional Information

**Accession codes**:The two crystal structures reported in this manuscript have been deposited at the Protein Data Bank (http://www.rcsb.org) as described in the text. PDB structure identifiers are 5J9B for wild type Asp f3 and 5J9C for the C31S/C61S mutant.

**How to cite this article**: Hillmann, F. *et al*. The Crystal Structure of Peroxiredoxin Asp f3 Provides Mechanistic Insight into Oxidative Stress Resistance and Virulence of *Aspergillus fumigatus. Sci. Rep.* **6**, 33396; doi: 10.1038/srep33396 (2016).

## Supplementary Material

Supplementary Information

## Figures and Tables

**Table 1:** X-ray crystallographic metadata for Asp f3.

	WT (5J9B)	C31S/C61S (5J9C)
Data collection		
Space group	P 2_1_2_1_2_1_	P 2_1_2_1_2_1_
Cell dimensions		
*a, b, c* (Å)	52.98, 68.49, 89.45	51.86, 68.10, 90.96
α, β, γ (°)	90.0, 90.0, 90.0	90.0, 90.0, 90.0
Resolution (Å)	31.98-2.10 (2.15-2.10)	30.56-1.96 (2.01-1.96)
Wilson B-factor	27.2	20.8
*R*_measured_	0.19 (0.95)	0.066 (0.31)
CC_1/2_	0.991 (0.631)	0.999 (0.992)
*I* / σ*I*	9.1 (2.0)	21.7 (4.4)
Completeness (%)	99.8 (100.0)	99.2 (90.8)
Redundancy	4.6 (4.5)	4.2 (2.9)
Refinement		
Resolution (Å)	2.10	1.96
No. reflections	19,598	23,739
*R*_work_ / *R*_free_	18.3/24.7	15.3/19.0
No. atoms		
Protein	2561	2662
Water	264	358
Metal ions	‒	1
*B*-factors		
Protein	23.4	18.6
Water	28.9	30.7
Metal ions	‒	42.5
R.m.s. deviations		
Bond lengths (Å)	0.007	0.008
Bond angles (°)	1.081	1.082
Ramachandran		
favored/allowed/disallowed	96.6/2.8/0.6	97.4/2.6/0.0

**Table 2:** Kinetic parameters of an Asp f3’s and its cysteine mutants’ enzymatic activities, determined by fluorometric and colorimetric assays with *t-*bOOH and H_2_O_2_ substrates.

Protein	*k*_*cat*_*, s*^−1^		*K*_*m*_*, M*^−6^		*k*_*cat*_*/K*_*m*_*, M*^−1^*s*^−1^ × 10^4^	
Fluorometric assay (Ampliflu Red)						
Asp f3	*t*-bOOH	H_2_O_2_	*t*-bOOH	H_2_O_2_	*t*-bOOH	H_2_O_2_
WT	12.96 ± 0.32	12.53 ± 0.89	70.48 ± 3.12	72.29 ± 5.12	18.39 ± 0.02	17.33 ± 0.12
C31A/C61A	4.92 ± 0.09	3.09 ± 0.08	86.77 ± 1.98	169.50 ± 7.45	5.67 ± 0.01	1.82 ± 0.02
C31S/C61S	1.91 ± 0.12	1.07 ± 0.07	32.01 ± 1.51	57.73 ± 1.65	5.97 ± 0.01	1.85 ± 0.07
Colorimetric assay (NADPH oxidation)						
WT	9.92 ± 0.34	10.45 ± 0.91	56.44 ± 6.26	65.04 ± 7.59	17.57 ± 1.34	16.07 ± 0.17
C31A/C61A	3.61 ± 0.09	1.91 ± 0.23	77.20 ± 9.35	148.9 ± 3.72	4.68 ± 0.95	1.28 ± 0.01
C31S/C6 1S	1.81 ± 0.15	1.05 ± 0.07	42.43 ± 2.52	77.20 ± 5.98	4.27 ± 0.89	1.36 ± 0.58
