# Supplementary material for: The Crystal Structure of Peroxiredoxin Asp f3 Provides Mechanistic Insight into Oxidative Stress Resistance and Virulence of Aspergillus fumigatus
Source: Sci Rep. 2016 Sep 14;6:33396. doi: 10.1038/srep33396 (PMC5022050; doi:10.1038/srep33396)
Supplement: Supplementary Information [file srep33396-s1.pdf]

Supplementary Information for:

The Crystal Structure of Peroxiredoxin Asp f3 Provides Mechanistic Insight into Oxidative Stress Resistance and Virulence of Aspergillus fumigatus

Short title: Aspergillus fumigatus Asp f3: Structure, Function, and Virulence

Falk Hillmann, Karine Bagramyan, Maria Strassburger, Thorsten Heinekamp, Teresa B. Hong, Krzysztof P. Bzymek, John C. Williams, Axel A. Brakhage, and Markus Kalkum

Supplementary Table S1 Interfacing residues < 3.6 Å

PISA output for WT

Salt bridges:

```
1 B:ARG 131[ NH1]  3.58  A:ASP  91[ OD2]
2 B:ARG 131[ NH2]  2.95  A:ASP  91[ OD2]
3 B:ASP  91[ OD2]  3.78  A:ARG 131[ NH1]
4 B:ASP  91[ OD2]  2.99  A:ARG 131[ NH2]
```

Hydrogen bonds:

```
1  B:ARG 131[ NH2]  2.95  A:ASP  91[ OD2]
2  B:THR  29[ O  ]  3.08  A:VAL  60[ N  ]
3  B:ALA  30[ O  ]  3.33  A:VAL  60[ N  ]
4  B:ALA  30[ O  ]  2.83  A:CYS  61[ N  ]
5  B:ASP  91[ OD2]  2.99  A:ARG 131[ NH2]
```

Hydrophobic contacts:

```
1 B:Ile 28 – A:Val 60
2 B:Phe 57 – A:Ala 97
3 B:Ala 97 – A:Phe 57
```

PISA output for C31/61S

Salt bridges:

```
1  B:ARG 131[ NH1]  3.14  A:ASP  91[ OD2]
2  B:ARG 131[ NH2]  2.80  A:ASP  91[ OD2]
3  B:ASP  91[ OD2]  3.35  A:ARG 131[ NH1]
4  B:ASP  91[ OD2]  3.10   A:ARG 131[ NH2]
```

Hydrogen bonds:

```
1  B:SER  31[ OG ]  3.04  A:PHE  57[ O  ]
2  B:ARG 131[ NH2]  2.80  A:ASP  91[ OD2]
3  B:TYR  89[ OH ]  2.69  A:ASP  91[ OD2]
4  B:PHE  57[ O  ]  3.02  A:SER  31[ OG ]
5  B:ASP  91[ OD2]  2.75  A:TYR  89[ OH ]
6  B:ASP  91[ OD2]  3.10   A:ARG 131[ NH2]
```

Hydrophobic Contacts:

```
1 B:Ile 28 – A:Ile 28
2 B:Phe 57 – A:Ala 89
3 B:Thr 58 – A:Tyr 93
4 B:Val 94 – A:Val 94
5 B:Ala 97 – A:Phe 57
```

Supplementary Table S2. Oligonucleotides used in the study.

Primer name	Sequence 5‘ to 3‘
WTf	TACGCCATGGGTTCTGGACTCAAGGCCGGTG
WTr	CGTAGCGGCCGCTTAGTGGTGGTGGTGGTGGTGCAGGTGCTTGAGGACGGTCTACGGTCT
C31Af	GGTGAGATCACTGCTGCCGGTATCCCCATCAAC
C31Ar	GTTGATGGGGATACCGGCAGCAGTGATCTCACC
C61Af	GCCTTCACTCCCGTCGCCTCTGCTCGCCACGTT
C61Ar	AACGTGGCGAGCAGAGGCGACGGGAGTGAAGGC
C31Sf	GGTGAGATCACTGCTTCCGGTATCCCCATCAAC
C31Sr	GTTGATGGGGATACCGGAAGCAGTGATCTCACC
C61Af	GCCTTCACTCCCGTCTCCTCTGCTCGCCACGTT
C61Ar	AACGTGGCGAGCAGAGGAGACGGGAGTGAAGGC
Aspf3-1	CAGCCCTCATACCCCATAAC
Aspf3-2	AGAAGATGAGGCGGGTGAG
Aspf3-3	TATATGCGGCCGCCGGGCCTCTAGCAAATAAACTT
Aspf3-4	ATATTACCCGGGGGCCTGAGTGGCCGCGGTCAATATATACACCATCTG
Aspf3-5	CATCAACTACAACGCCTCCA
Aspf3-6	GGAGGTGAGAAACGAGTTGG
Aspf3-7	ATATTAAAGCTTGGCCATCTAGGCCCCTTCACTCCCGTCTGCTCT
Aspf3-8	TATCGTTAATTAATGCCGAACAGACGGATTATT
Aspf3C-fw	AAAACCCGGGAGAATTCGAGAAAGCGAG
Aspf3C-rv	GAGTCCCGGGATACCACTAATG
LF-ASPF3-fw	CAATCAATTGCCAGTTTGC
LF-ASPF3-rv	GCGGTAAGCTAGAGTAGTGG

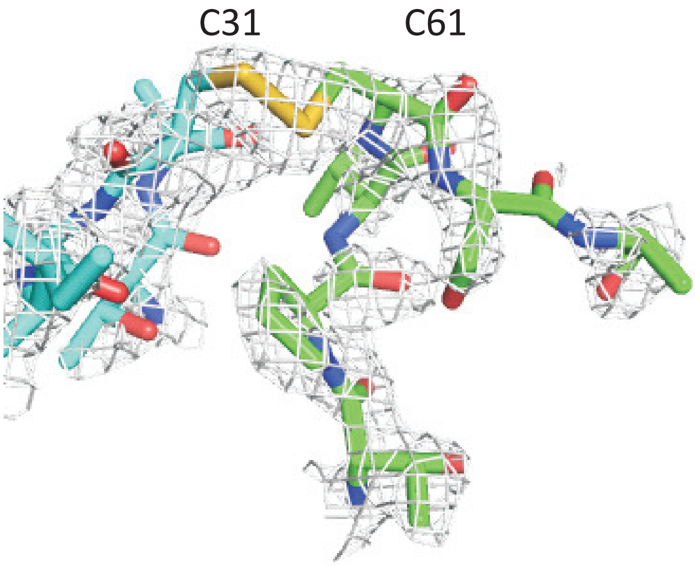

Supplementary Fig S1. Stereo image of simulated annealing composite omit map around C31-C62 residues of WT Asp f3 contoured at σ=1.0.

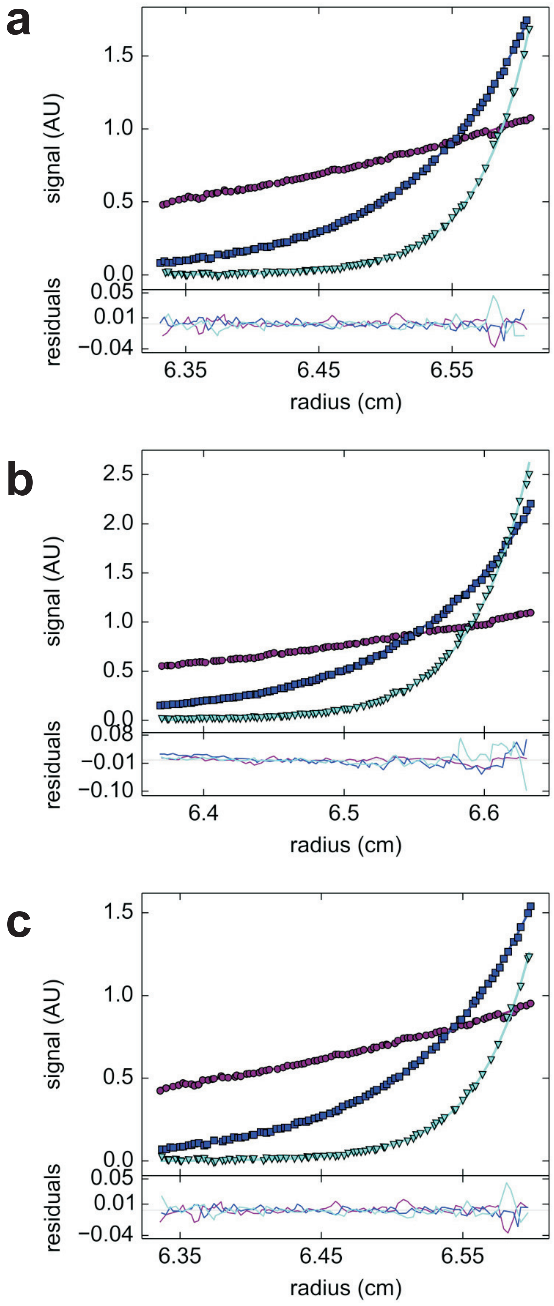

Supplementary Fig S2. Aspf3 sedimentation equilibrium experiments performed in PBS at 25°C. Data was collected after 10 h equilibration at 10,000 rpm (purple), 20,000 rpm (blue) and 30,000 rpm (cyan). Protein concentration was 10 μM. (**a**) WT Aspf3; (**b**) WT Aspf3 in the presence of 1 mM TCEP; (**c**) C31S/C61S Aspf3 variant. Data was fit using a monomer-dimer association model; The calculated molecular weight of the associating species ranged from 35.5 to 37.3 kDa, consistent with a dimer.

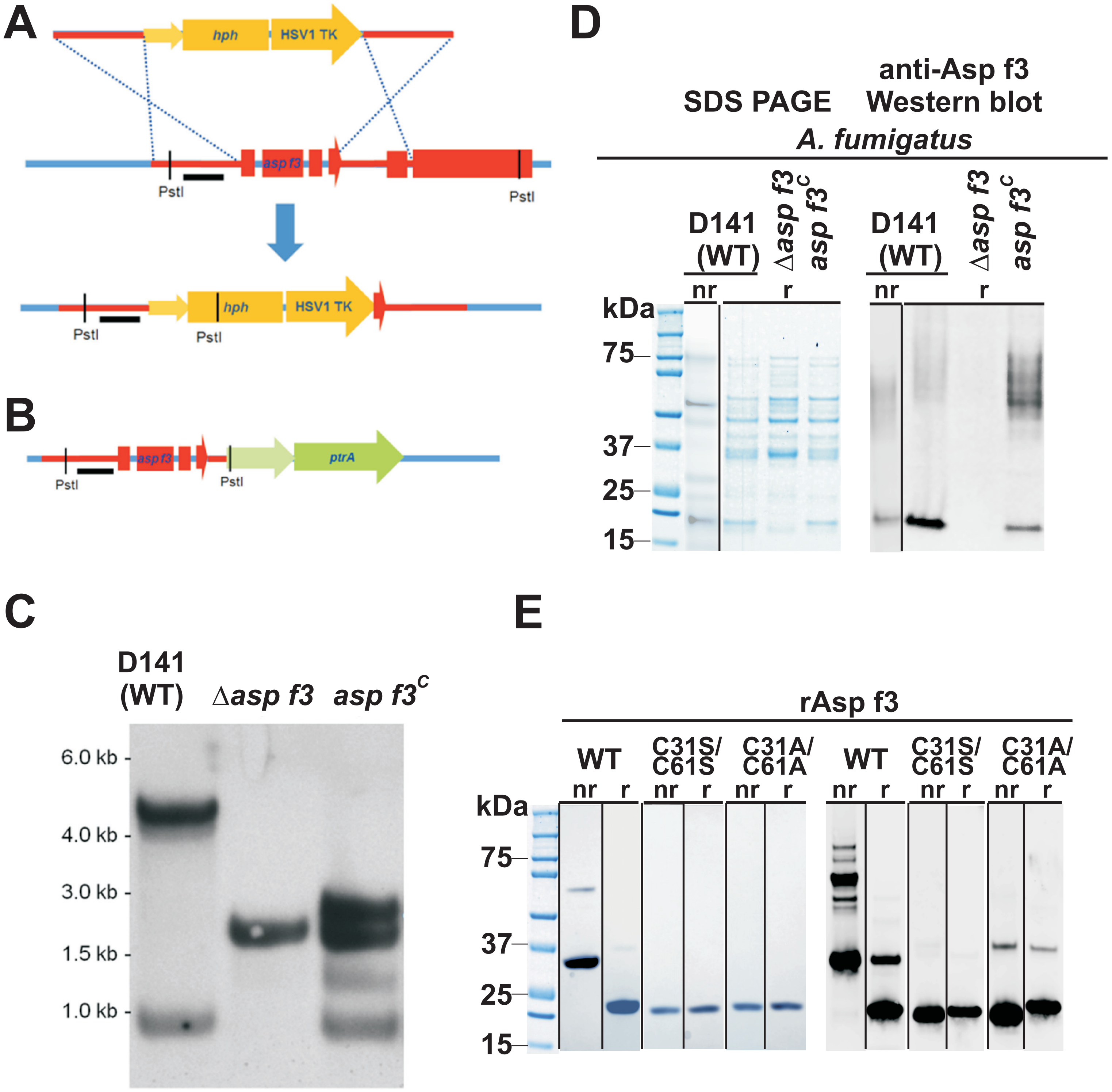

Supplementary Fig S3. Gene deletion of *asp f3* in *A. fumigatus*. (A) Deletion mutants were generated by homologous recombination using a hygromycin resistance cassette with upstream and downstream DNA sequences of the *asp f3* encoding gene (AFUA_6G02280). (B) Deletion mutants were complemented with the *asp f3* gene locus linked to the pyrithiamine resistance cassette. Homologous recombination and chromosomal integration of the gene cassettes were verified by (C) Southern hybridizations using probes directed against the upstream region of *asp f3* and the absence of the Asp f3 protein in the deletions strains was further confirmed by (D) Coomassie stained SDS-PAGE and Western blot with *A. fumigatus* cell lysates from the following strains: parental D141 (WT), deletion (Δ*asp f3*), the complemented strain (*asp f3*^{C}), and (E) with recombinant Asp f3 and its mutants under reducing (r) and non-reducing (nr) conditions. (D-E) Lanes were rearranged according to the loading order and demarcated by black lines.

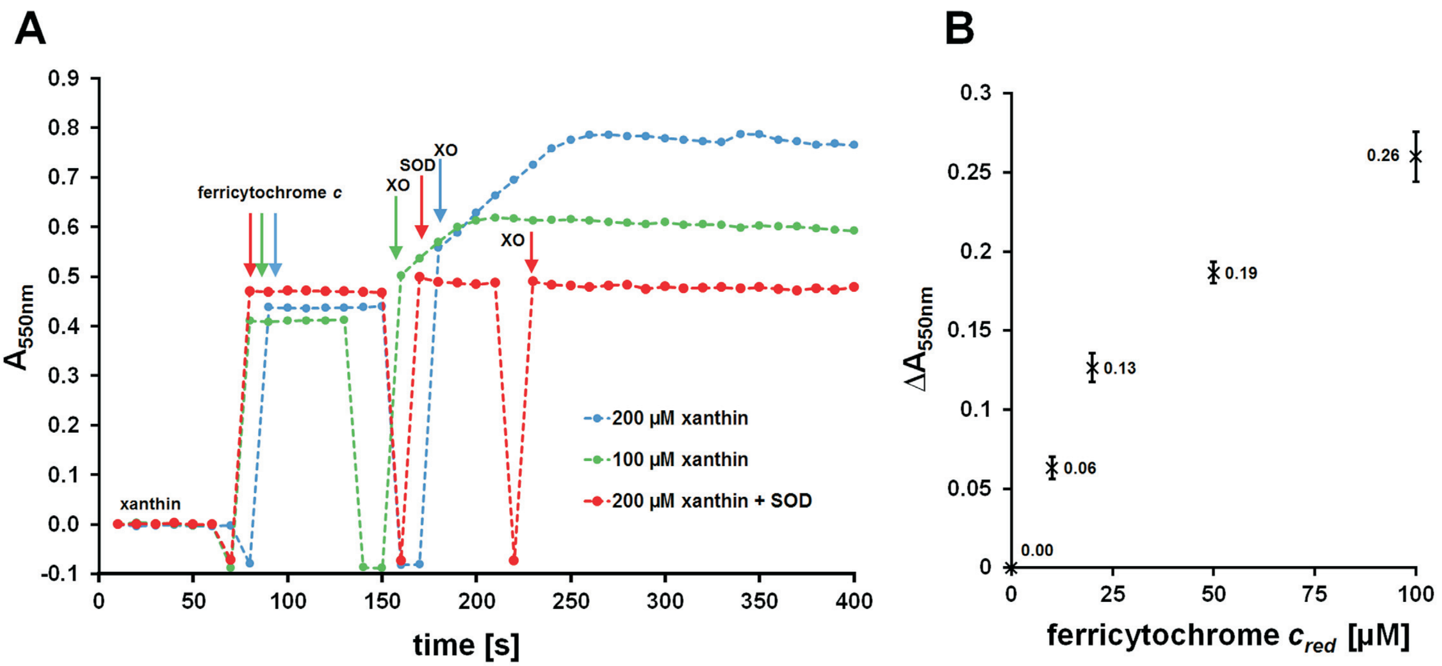

Supplementary Fig S4. **Enzymatic production and quantification of $\cdot O_2^{-}$ by the ferricytochrome reduction assay [20].**

A) Xanthine oxidase (100 µg/mL) was added to Czapek-Dox medium with varying concentrations of xanthin and 100 µM ferricytochrome *c* (Sigma-Aldrich**, C3131**). The reaction was followed at 37°C as an increase in absorbance at λ=550 nm. The $\cdot O_2^{-}$-dependent reduction of ferricytochrome *c* was strictly dependant on the xanthine concentration and was fully inhibited by the addition of SOD (red line) at 20 units/mL (Sigma-Aldrich, S4636). Data were acquired from three independent experiments and one representative data set is displayed. 200 µM (blue line) and 100 µM of xanthine (blue line) led to a linear increase in absorbance ($A_{550\ nm}$) from 0.45 to 0.75 and from 0.41 to 0.60, respectively. **B)** The absorbance difference between oxidized and completely, dithionite-reduced ferricytochrome *c* ($\Delta A_{550\ nm}$) depends on the total concentration of ferricytochrome *c*. The standard curve demonstrates that xanthine oxidation and $\cdot O_2^{-}$ release occurred at a molar ratio of approximately 2:1.

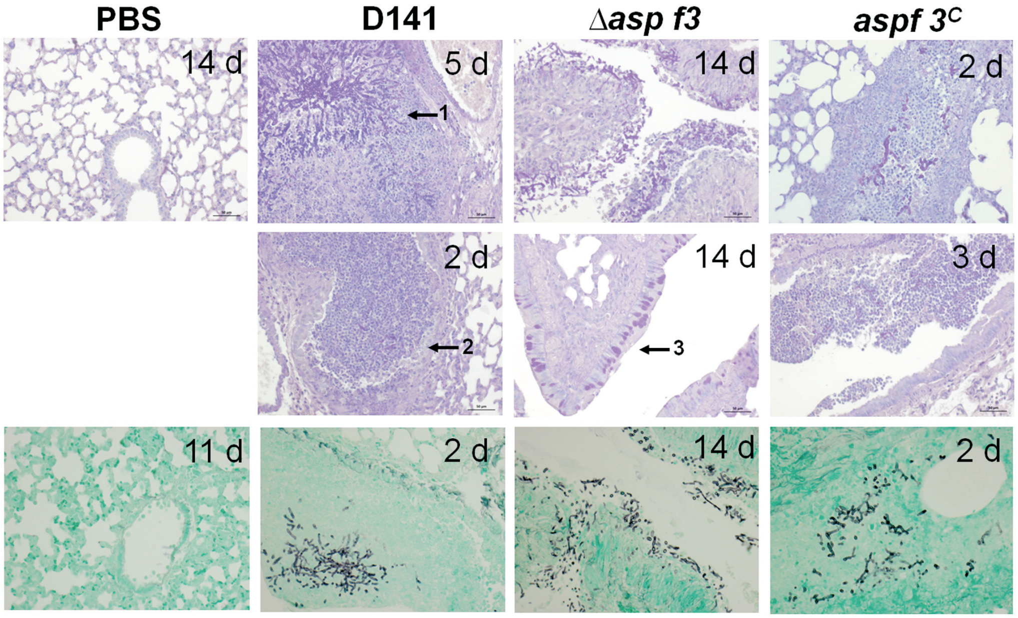

Supplementary Fig S5. **Histology of lungs from mice infected with *A. fumigatus*.** Lungs were fixed in buffered formalin and embedded in paraffin; 4-µm sections were stained using Periodic acid-Schiff (PAS, hyphae stain magenta) or methenamine silver (hyphae stain black). The presence of invasive fungal hyphae (magenta or black), destructed lung tissue, and infiltration of immune cells (purple nuclei) was confirmed in lungs of mice infected with WT *A. fumigatus* (D141) and the complemented strain (*asp f3*^{C}) 2-5 days post infection. Invasive hyphae (arrow 1) and infiltration of immune cells (arrow 2) were detected in lung sections infected with the WT, but comparably few immune cells or invasive hyphae were detected 14 d after infection with the deletion mutant (Δ*asp f3*). Occasional goblet cell hyperplasia was observed (arrow 3). A lung section of a sham-infected mouse is shown as a control (PBS).

```
                                  CR                             CP
                                  ↓                              ↓
Asp f3   10 FPS-DVVFSYIPWSEDKGEITACGIPINYNASKEWA-DKKVILFALPGAFTPVCSARHVP  67
            FP+ D  F YI  S+   +  +C +P     SK  + +KKVI+   P AF+P C+  H+P
Ahp  1    9 FPAGDYKFQYIAISQSDADSESCKMPQTVEWSKLISENKKVIITGAPAAFSPTCTVSHIP  68

Asp f3   68 EYIEKLPE-IRAKGVDVVAVLAYNDAYVMSAWGKANQVTGDD-ILFLSDPDARFSKSIGW  125
             YI  L E ++ K VD V V+  ++ +   AW K+  V     I F SDP   F+KSIG+
Ahp  1   69 GYINYLDELVKEKEVDQVIVVTVDNPFANQAWAKSLGVKDTTHIKFASDPGCAFTKSIGF  128

Asp f3  126 ADEEGR----TKRYALVIDHGKITYAALEP-AKNHLEFSSAETVLKHL              168
                G     + R+A+V+++G +TYAA E      +  SS E+VL HL
Ahp  1  129 ELAVGDGVYWSGRWAMVVENGIVTYAAKETNPGTDVTVSSVESVLAHL              176
```

Supplementary Fig S6. Amino acid sequence alignment of *A. fumigatus* Asp f3 and *S. cerevisiae* Ahp1. The position of the peroxidatic (C_P) and resolving (C_R) cysteine residues are indicated by arrows. The catalytic active-site motif (**PXXXS/TXXC**) is conserved in both proteins.

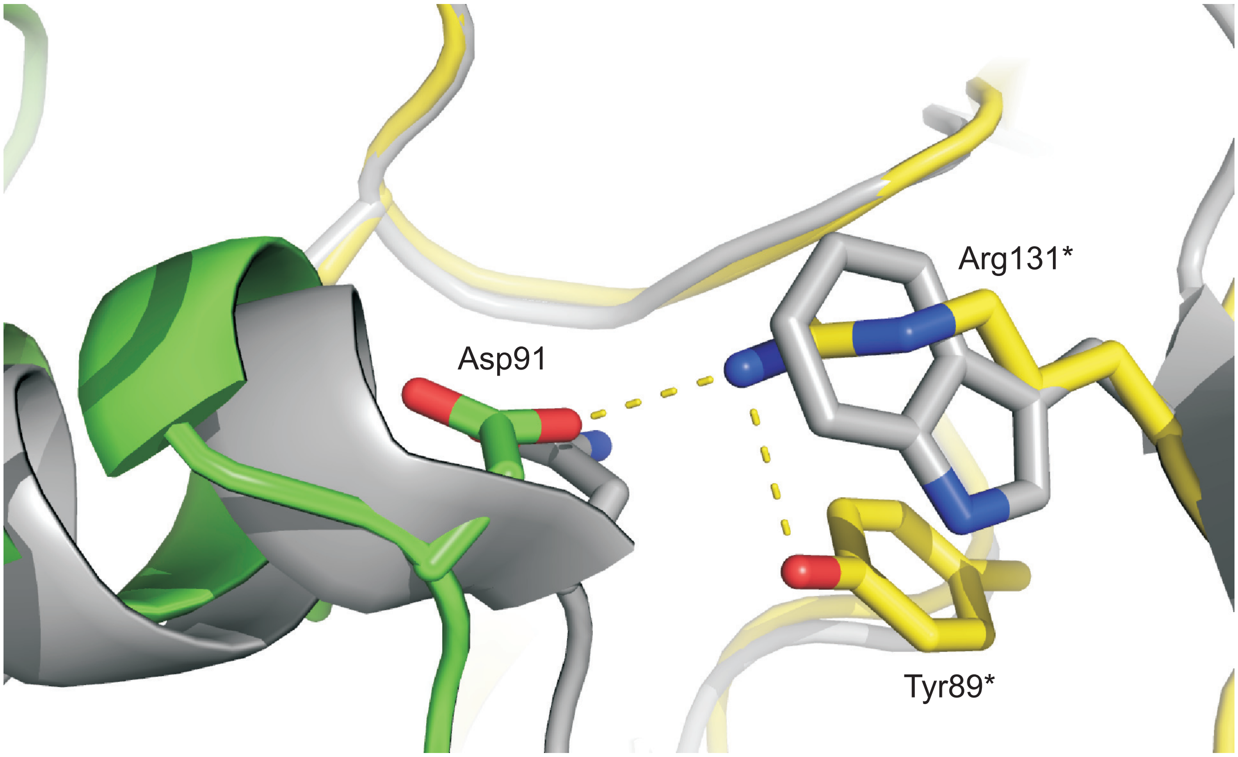

Supplementary Fig S7. A structural difference between the dimerization interfaces of Asp f3 (*A. fumigatus*) and Ahp1 (*S. cerevisiae*). Asp f3 backbones are shown in green and yellow, Ahp1 backbones in grey; Unique to Asp f3: the carboxylate in Asp91 in makes a backbone h-bond (distance = 2.9 Å), capping the helix. Arg131* from the other protomer stabilizes the Asp91 through a salt bridge (distance = 3.0 Å). Moreover, the hydroxyl group of Tyr89* makes an H-bind to the guandinium group of Arg131* (3.3 Å). This part of the interface is not affected by the large conformation change that involves the redox active cysteines. The asterisk marks residues of the opposite protomer.

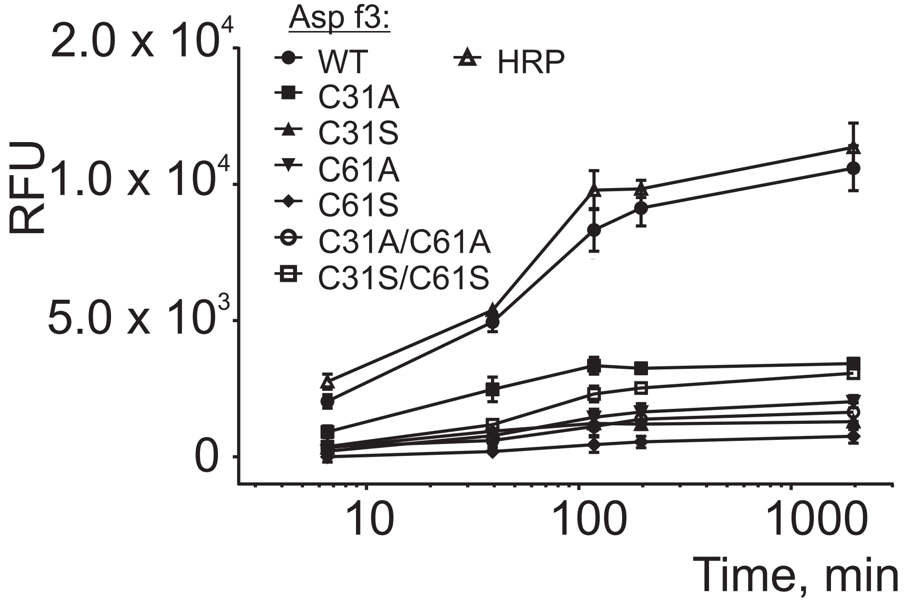

Supplementary Fig S8. Time-based kinetic analysis of fluorescence produced by Asp f3 WT and its mutants with AmplifluRed and H_2O_2. Fluorescent measurements were made at 535/585 Excitation/Emission wavelengths. HRP was used as a positive control. Data represents the average of three independent measurements.

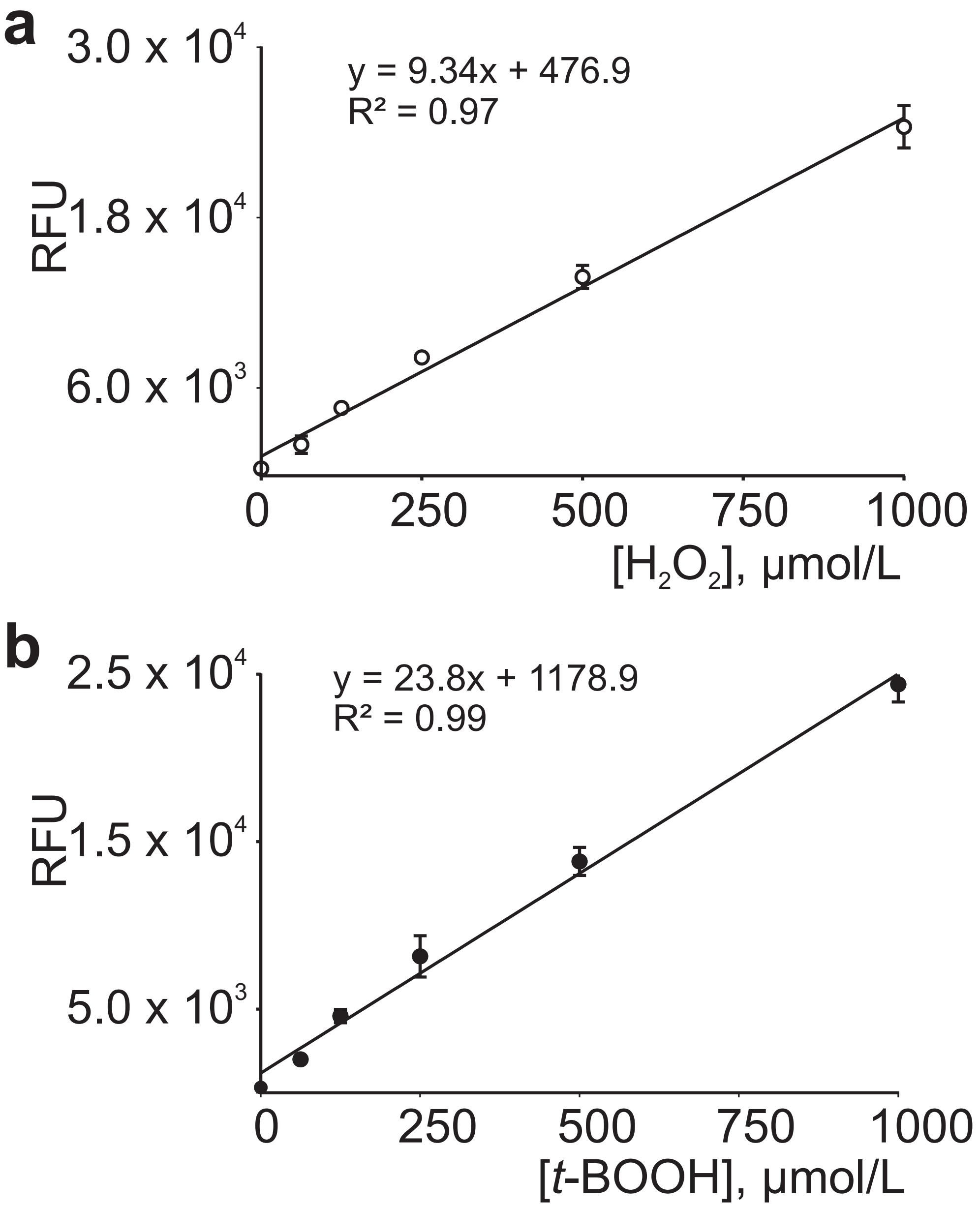

Supplementary Fig S9. Standard curves generated with an HRP. Each reaction contained Ampliflu Red and (**a**) H_2O_2 or (**b**) *t*-bOOH ranging in concentration from 0 to 1 mM. The data was fitted by linear regression analysis represented by the inserted equations.
